# Supplementary material for: Multidimensional diffusion processes in dynamic online networks
Source: PLoS One. 2020 Feb 6;15(2):e0228421. doi: 10.1371/journal.pone.0228421 (PMC7004299; doi:10.1371/journal.pone.0228421)
Supplement: S1 File — (PDF) [file pone.0228421.s001.pdf]

Multidimensional diffusion processes in dynamic online networks - Supporting information

David Easley [1◑], Eleonora Patacchini [1◑], Christopher Rojas* [1◑],

1 Department of Economics, Cornell University, Ithaca, NY, USA

◑These authors contributed equally to this work.
* car332@cornell.edu

A1: Background on GitHub

GitHub is an online platform for collaborative software development, based on the Git version control system. On GitHub, users *create* software projects known as repos. Each repo is a directory which holds all of the files associated with the project, such as source code and documentation files. The repo exists both locally on a user's machine, and remotely on GitHub. The user makes changes to the repo on her local machine, such as adding code, and then *pushes* the changes to the repo on GitHub, so that the online repo matches her local copy. The user who creates the repo also designates a list of collaborators; these are other users who can push changes to the content of the repo. If the repo is public, then the collaborators are not the only users who can contribute to the repo. In fact, any user on GitHub can *fork* a public repo to create a copy of the repo with which they can freely experiment. The user can then push changes to their forked copy without affecting the original repo. The forked repo can be the starting point for a new independent project, or it can be used to propose changes to the original repo. If a user wishes to merge the changes in her forked repo back into the original repo, and she is not a collaborator, then she can submit a *pull* request. Any of the collaborators may decide whether to approve or reject the requested changes. If approved, then those changes are merged back into the original repo.

However, GitHub is more than just a code hosting service. It also offers social networking features to help users find interesting projects and to support the community of developers. Note that at the time of our study, GitHub did not have a recommendation system to help users find interesting projects. Users can *star* interesting repositories that they want to bookmark for later reference, and/or to show their appreciation to the project's developer(s). The total number of forks and stars of a repo are prominently displayed on the repo's GitHub page. In addition to that, GitHub users can *follow* other users, to be notified of some of their activities. GitHub users can follow other users without the other user's consent, similar to following someone on Twitter. Once a following relationship is established, the follower will receive notifications on her GitHub homepage when the leader creates, forks, or stars a repo, when the leader is added as a collaborator to a repo, and when the leader follows another user. However, the leader will not receive notifications about the follower. Every GitHub user has a profile page with optional information about the user, such as the company for which the user works and her physical location. The user's profile page includes information about the public repos that the user has created, forked or starred, and the contributions to public repos which she has made. The user's profile page also lists her total number of followers and leaders, and links to the profile pages of all of the user's followers and leaders.

Besides its public open-source repositories, GitHub also sells private repos which can only be accessed by collaborators, and it sells on-premise instances of its software for businesses. However, we will focus exclusively on public repos, because these are the repos for which we have data, and more importantly, they are the repos for which GitHub's social networking features are relevant.

Like many online networks, GitHub is popular with young people, but it has some unique features. We do not have detailed demographic information for most of the users in our main dataset, but a recent survey of GitHub users can tell us about the types of individuals who currently use GitHub, which is likely similar to the users in our data [1]. The survey results suggest that the overwhelming majority of GitHub users are men, with 90.95% of survey respondents identifying as male and only 3.36% identifying as female. This is similar to the collaborative content creation sites Wikipedia and StackOverflow. GitHub users are young, with over 63.45% of respondents being under 34 years old. They are also well-educated, with 65.7% of respondents having at least a bachelor's degree. Results from [2], which were collected in 2011-2012, show that while GitHub users are located all over the world, the majority of users are in North America or Western Europe, with the U.S.A. having the largest fraction at 30.14%. Aside from gender, the demographics of GitHub users are similar to those found in other online networks targeting young people. GitHub is also a very prominent technology company, used by companies including Google, Facebook, Twitter and Microsoft (and many others) to house their open-source software projects and to reach out to the developer community. As of early June, 2018, Github had over 27 million active users ([3]), and according to the website traffic monitor Alexa, it is the 60th most popular website on earth.

The content creation patterns on GitHub are broadly similar to what has been observed in other online social networks. The so-called "90-9-1" rule is a commonly observed pattern of online user-generated content distributions, in which 90% of users do not contribute anything, 9% of users occasionally create content and 1% of users drive a large amount of the activity [4]. In our data we cannot see the users who do not contribute anything, but we can see that a small fraction of very active users create a large fraction of the content. For instance, when we look at the 1,763,923 unique users in our data who created at least one repo, 46.31% of the repos are created by the top 10% of the users. For the 785,584 users who starred at least one repo, the top 10% of users were responsible for 73.5% of the stars. The remaining behaviors in we analyze in this paper fall in between these two extremes. Compare these results to other platforms such as Wikipedia, in which the top 2.5% of users contribute 80% of the content [5], and Twitter, in which the top 10% of users write 90% of the Tweets [6]. If anything, the distribution of behaviors is less skewed on GitHub than on these other platforms.

However, there are important differences between GitHub and more typical online social networks primarily about interaction, such as Twitter. Many GitHub users do not even participate in the social network at all; for instance, of the GitHub users in our data who created at least one repo, only 31.54% are involved in the social network at all. For users in the social network graph, their average degree of 3.65 is much lower than on Twitter [2]. Also, only 9.6% of the pairs of connected users in our social network have a reciprocal relation between them, while the rest are one-sided. This is again much lower than the rate on Twitter of 22% [2]. These numbers support the idea that the social network is less central to the purpose of GitHub than it is for Twitter, and that following users on GitHub can be more costly in the sense that following many other developers on GitHub results in receiving many notifications from them, which can be distracting. Even though the social network is less central to GitHub's purpose, it still is large and plays significant role in connecting the most active users; the 860,071 users involved in the social network generate the majority of creates, forks, stars and pushes

in our data.

A2: Maximum posterior weighted-likelihood based on the WRMF model

We will infer estimates of preferences based on the statistical model used by the popular collaborative filtering algorithm referred to as WRMF ([7]). The model is static and there is no social network. Let m_t denote the number of items on the platform by the end of period t, and let n_t denote the number of agents who belong to the platform by the end of period t. The adoption matrix $\mathbf{Y}_t = \{Y_{u,i,t}\}$ at the end of period t is the $n_t \times m_t$ matrix with a 1 in its (u, i)-th position if agent u has adopted item i by the end of period t, and zero otherwise. We assume that at the end of period t, each agent u has linear preferences over characteristics of items, represented by (N-dimensional vector) $\mathbf{W}_{u,t}$, and that each item has characteristics represented by (N-dimensional vector) $\mathbf{V}_{i,t}$. Hence, the utility for agent u from adopting item i would be $\mathbf{W}_{u,t} \cdot \mathbf{V}_{i,t}$. Let $\mathbf{W}_t, \mathbf{V}_t$ denote the matrices of stacked row vectors for $\mathbf{W}_{u,t}, \mathbf{V}_{i,t}$, respectively.

WRMF is estimated at the end of period t by using only the adoption matrix; the algorithm assumes that the adoption of item i by agent u by the end of period t is based on the following equation:

$$Y_{u,i,t} = \mathbf{W}_{u,t} \cdot \mathbf{V}_{i,t} + \mu_{u,i,t}, \quad \mu_{u,i,t} \sim N(0, \sigma_\mu) \tag{A5}$$

Note that WRMF assumes that agents have a negative preference towards any items which they have not adopted, but we can choose how confident we are in that assumption through the use of confidence weights. Let $C_{u,i,t}$ denote the (integer) confidence weight for agent u and item i. WRMF assumes that the confidence weights take the following form:

$$C_{u,i,t} = 1 + \alpha Y_{u,i,t} \tag{A6}$$

We incorporate the confidence weights by letting each observation serve as $C_{u,i,t}$ independent observations. By assuming that all of the observations are independent, we can derive the weighted likelihood of our observations $\mathbf{Y}_t$, given the parameters $\mathbf{W}_t, \mathbf{V}_t$:

$$\mathcal{L}(\mathbf{Y}_t|\mathbf{W}_t, \mathbf{V}_t) = \prod_{u,i} g(Y_{u,i,t}|\mathbf{W}_{u,t}, \mathbf{V}_{i,t})^{C_{u,i,t}} \tag{A7}$$

g is the probability density function for a normal distribution. Note that the data is binary but the distribution of our unobservable is normal; there are other versions of collaborative filtering that use a logistic function to model the binary outcomes. WRMF assumes zero-mean spherical Gaussian priors for the preference and characteristic vectors:

$$\mathbf{W}_{u,t} \sim N\left(\mathbf{0}, \tilde{\rho}^{-1}\mathbf{I}_N\right), \quad \mathbf{V}_{i,t} \sim N\left(\mathbf{0}, \tilde{\rho}^{-1}\mathbf{I}_N\right). \tag{A8}$$

We take the log of the posterior weighted likelihood and replace constant terms with λ to get the following:

$$\ln \mathcal{L}\left(\mathbf{W}_t, \mathbf{V}_t|\mathbf{Y}_t\right) = -\sum_{u,i} C_{u,i,t}\left(Y_{u,i,t} - \mathbf{W}_{u,t} \cdot \mathbf{V}_{i,t}\right)^2 - \lambda\left(\sum_u ||\mathbf{W}_{u,t}||_2^2 + \sum_i ||\mathbf{V}_{i,t}||_2^2\right). \tag{A9}$$

We learn $\mathbf{W}_t, \mathbf{V}_t$ to maximize the posterior weighted likelihood.

A3 Appendix: WRMF estimation by alternating least squares

Solving the log-likelihood equation for WRMF (Section 6.2) for a global maximum is not feasible with large datasets. Instead, the values of $\mathbf{W}_t, \mathbf{V}_t$ can be found by alternating least squares, because when we fix either $\mathbf{W}_t$ or $\mathbf{V}_t$ the equation becomes quadratic and so its maximum can be easily computed. To do alternating least squares, begin by randomly guessing values for $\mathbf{W}_t$, and then solve for the $\mathbf{V}_t$ that maximizes the log-likelihood. Then, fix the values of $\mathbf{V}_t$ at what was just found, and solve for the $\mathbf{W}_t$ that maximizes the log-likelihood. Repeat this process a pre-specified number of times, which we will denote by A. α, λ, N and A are all different hyper-parameters which must be set before we can do alternating least squares.

The models in this paper were estimated using the implementation of alternating least squares available in the repo *benfred/implicit* on GitHub ([8]).

A4: Details of hyper-parameter setting

The statistic we compute to set the hyper-parameters is known as the Discounted Cumulative Gain, or DCG ([9]). Let $\text{Rank}(u, i)$ denote the predicted ranking of an item adopted in the test set. We compute the following:

$$\text{DCG} = \sum_{u,i} \frac{1}{\log_2(\text{Rank}(u, i))} \tag{1}$$

The reason we favor this statistic is because it places more emphasis on getting rankings near the top correct. We drop any observations in the test set with a predicted ranking greater than 100. The objective in selecting hyper-parameters is to maximize the value of this statistic.

Preliminary tests revealed that a greater number of factors tends to lead to a higher value of the statistic. Hence, to reduce the number of hyper-parameters over which we need to search, we fixed the number of latent factors at the largest value we tried, $N = 100$. We also fixed the number of alternations at $A = 15$. We then tested random values of the hyper-parameters α, λ. Each value was drawn from a uniform distribution over the set of integers $\{1, ..., 1000\}$. We tested 120 pairs of values. In the end, we use the values $\alpha = 95, \lambda = 864$, which results in $\text{DCG} = 0.468$

A5: Incorporating exposure into WRMF

We include a robustness check in which we modify WRMF to incorporate past exposure. For learning preferences in period t, define the exposure indicator $E_{u,i,t}$ as a variable which is equal to 1 if agent u would have been treated in the past on item i, according to our definition of treatment (Section 2.1), and if the treatment expired by the end of period t. In other words, we have the following:

$$E_{u,i,t} = \begin{cases} 1, & \text{if } Y_{u,i,t} = 0, \quad T_{u,i,s} = 1 \text{ for some } s < t - 3, \quad T_{u,i,t} = 0 \\ 0, & \text{otherwise} \end{cases}$$

We generalize $C_{u,i,t}$ to be the following:

$$C_{u,i,t} = \begin{cases} 1, & \text{if } Y_{u,i,t} = 0, \quad E_{u,i,t} = 0 \\ 1 + \alpha_1, & \text{if } Y_{u,i,t} = 0, \quad E_{u,i,t} = 1 \\ 1 + \alpha_2, & \text{if } Y_{u,i,t} = 1 \end{cases}$$

We can now adjust the confidence in the case where an agent has not adopted a given item, but was treated on the item sufficiently long in the past. We use a value of $\alpha_1 > 0$, so that if agent u's leaders adopted the item, then we are more confident that a negative preference revealed by agent u for item i ($Y_{u,i,t} = 0$) is measured correctly. We also assume that $\alpha_1 <= \alpha_2$, since we don't know for certain if an agent was exposed to an item whenever the revealed preference is negative.

A6: Pigeonhole bootstrap

We include as a robustness check an alternative bootstrap technique, known as the Pigeonhole bootstrap, which accounts for two-way random effects ([10], [11]). We do bootstrap re-sampling on the matched pairs, as before. However, for each of 100 bootstrap replicates, we re-weight observations according to the following procedure. For a particular replicate, each agent is assigned the outcome of a Bernoulli($p = 0.5$) draw, and each item is also assigned the outcome of a Bernoulli($p = 0.5$). Each observation is then assigned the product of the draws for the agent and item of that observation as its weight. Hence, an agent-item pair appears in a bootstrap replicate if and only if both the agent and the item are in the replicate.

Note that [12] show that matching estimators do not satisfy the required smoothness conditions for consistency of the bootstrap. However, the Pigeonhole bootstrap is known to produce confidence intervals that are too large in a setting with two-way (agent, item) random effects ([10], [11]), and in our robustness checks we show that our main result is still significant even with the Pigeonhole bootstrap.

A7: Additional Tables and Figures

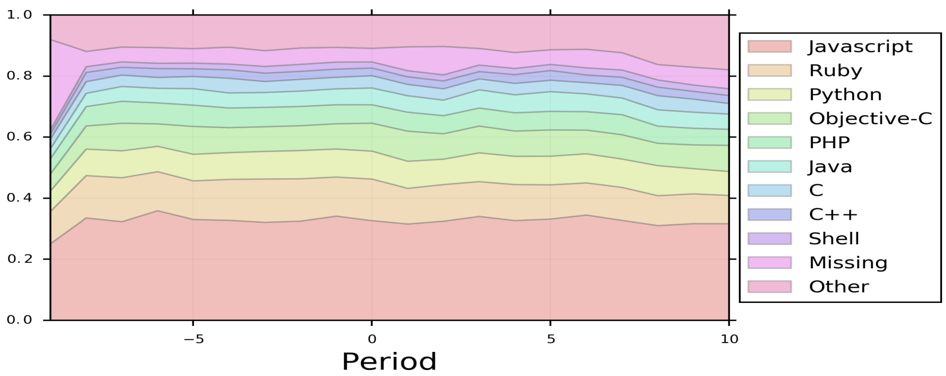

Fig 1. The fraction of stars each period (beginning in period -9) of repos from various programming languages. Missing means that the language field is empty, and Other means that the language is some other language besides the ones listed.

Table 1. Summary statistics for the distributions of stars and follows per agent/repo (A) and per period (B).

(A)

	Stars Per Agent	Stars Per Repo	Follows Per Agent
Count	163,458	855,317	163,458
Mean	61.40	11.73	10.17
Std. Dev.	146.31	119.78	114.70
Min.	10	1	0
25%	16	1	1
50%	28	1	4
75%	60	3	10
99%	510	179	87
Max	28,148	37,959	38,541

(B)

	Stars Per Period	Follows Per Period
Count	33	33
Mean	304,151.12	50,375.55
Std. Dev.	151,236.57	19,769.92
Min.	67,443	5,724
50%	292,075	53,213
Max	537,477	93,400

Table 2. The demographic and behavioral covariates used for nearest-neighbor matching, aside from previous adoption behaviors.

Characteristic Type	Variable
Personal	Experience (Months)
Behavior	Number of followers
	Number of followees
	Number of Repos Starred
Average leader Personal	Average leaders' experience
Average leader behavior	Average leaders' number of followers
	Average leaders' number of followees
	Average leaders' number of repos starred

We use logarithms (log + 1) of all covariates except for experience.

Table 3. The demographic and behavioral covariates used for estimating homophily.

Characteristic Type	
Personal	Experience (Months)
Behavior	Number of followers
	Number of followees
	Number of Repos Starred
	WRMF Agent Preferences

We use logarithms (log +1) of number of followees, number of followers, and starred repos. All of the covariates are normalized before estimating homophily.

References

1. GitHub. Open Source Survey; 2017. Available from: `http://opensourcesurvey.org/2017/`.

2. Lima A, Rossi L, Musolesi M. Coding together at scale: GitHub as a collaborative social network. In: ICWSM; 2014. p. 295–304.

3. Heater B. Microsoft is reportedly acquiring GitHub; 2018. Available from: `https://techcrunch.com/2018/06/03/microsoft-is-reportedly-acquiring-github/`.

4. Ochoa X, Duval E. Quantitative Analysis of User-Generated Content on the Web. In: Proceedings of the First International Workshop on Understanding Web Evolution (WebEvolve2008). Beijing; 2008. p. 19–26.

5. Tapscott D, Williams A. Wikinomics: How Mass Collaboration Changes Everything. Portfolio Hardcover; 2008.

6. Heil B, Piskorski M. New Twitter Research: Men Follow Men and Nobody Tweets; 2009. Available from: `https://hbr.org/2009/06/new-twitter-research-men-follo`.

7. Hu Y, Koren Y, Volinsky C. Collaborative Filtering for Implicit Feedback. In: IEEE International Conference on Data Mining; 2008. p. 263–272.

8. Fredericks B. Implicit: Fast Python Collaborative Filtering for Implicit Feedback Datasets; 2018. Available from: `https://github.com/benfred/implicit`.

9. Järvelin K, Kekäläinennd J. Cumulated Gain-based Evaluation of IR Techniques. ACM Transactions on Information Systems. 2002;20.

10. Owen AB. The Pigeonhole Bootstrap. 2007;1(2):386–411. doi:10.1214/07-AOAS122.

11. Owen AB, Eckles D. Bootstrapping Arrays of Arbitrary Order. The Annals of Applied Statistics. 2012;6(3):895–927. doi:10.1214/12-AOAS547.

12. Abadie A, Imbens GW. On the Failure of the Bootstrap for Matching Estimators. Econometrica. 2008;76(6):1537-1557. doi:10.3982/ECTA6474.
